# Are Plasma IL-10 Levels a Useful Marker of Human Clinical Tolerance in Peanut Allergy?

**DOI:** 10.1371/journal.pone.0011192

**Published:** 2010-06-17

**Authors:** Larisa C. Lotoski, F. Estelle R. Simons, Rishma Chooniedass, Joel Liem, Isha Ostopowich, Allan B. Becker, Kent T. HayGlass

**Affiliations:** 1 Department of Immunology, University of Manitoba, Winnipeg, Manitoba, Canada; 2 Department of Pediatrics and Child Health, University of Manitoba, Winnipeg, Manitoba, Canada; 3 Windsor Allergy Asthma Education Centre, Windsor, Ontario, Canada; New York University, United States of America

## Abstract

**Background:**

Food allergies are a major component of the burden of allergic disease. Accurate risk assessment for prediction of future clinical reactivity or clinical tolerance is limited by currently available techniques. Recent studies suggest that constitutively elevated global serum levels of IL-10, a cytokine that down-regulates both Th1 and Th2 cytokine production, may be useful in identifying human clinical tolerance to foods.

**Objective:**

Determine the usefulness of constitutive IL-10 levels as a marker of clinical tolerance to peanut in children and adults.

**Methodology/Principal Findings:**

107 subjects who were clinically tolerant to peanut and 94 subjects who were clinically allergic to peanut participated. Plasma was analyzed via ELISA to quantify the frequency of individuals with constitutive IL-10 levels and the intensity of those responses. The data were then stratified by age, gender and clinical status to assess the utility of this putative biomarker in specific at-risk groups. All 201 subjects had readily quantified plasma IL-10. Levels were no higher in subjects who were clinically tolerant to peanut than those in individuals clinically allergic to peanut. Stratification by age, gender or both did not improve the capacity of IL-10 levels to identify clinical tolerance to peanut.

**Conclusions/Significance:**

Plasma IL-10 levels are neither a useful biomarker of clinical tolerance to peanut nor a potential tool for identification of clinical tolerance to peanut in humans.

## Introduction

Peanut allergy is a leading cause of fatal anaphylaxis [1] and affects approximately 1.2 to 1.5% of people in North America and the United Kingdom [2], [3]. The methods most commonly used for risk assessment include a detailed medical history of symptoms after peanut exposure, a skin prick test to peanut and quantification of peanut specific IgE in serum [4]. These tests are used in combination, as there is the potential for diagnostic error with reliance on any single method in isolation. Skin prick tests or peanut specific IgE levels are unable to differentiate reliably between sensitization to peanut, which is common, and risk of systemic allergic reaction to peanut, which is considerably less common [5]–[7]. While time consuming, costly and potentially risky, double blind placebo controlled food challenges (DBPCFC) are the gold standard for identification of clinical reactivity to peanut or other foods. However, DBPCFC are performed under standardized conditions in a healthcare setting and are not 100% predictive of clinical reactions in the community. In addition, the severity of clinical reactions experienced upon accidental exposure in the community does not necessarily correlate well with the severity as assessed during low dose allergen challenge in the clinic [6], [8].

Erroneously classifying patients as low risk for clinical reactivity to peanut presents obvious problems, while erroneously classifying them as high risk based on low or moderate sensitization to peanut leads to a negative psychosocial impact and reduced quality of life [9], [10]. Given that the prevalence of patients with clearly positive skin prick tests to peanut is some 10-fold higher than those with verifiable clinical peanut allergy [11], there is great interest in development of new quantitative tools to complement current methods of peanut allergy assessment. No single biomarker with sufficient sensitivity and specificity is currently available for identifying clinical reactivity to peanut.

Identification of novel biomarkers that would reliably allow determination of clinical status and/or disease severity is a major focus in many areas of biomedical research. Most of this work has concentrated on identification of biomarkers that associate with disease pathogenesis or exacerbation rather than clinical tolerance. Markers with sufficient sensitivity and specificity to offer clinical utility have been sought for cancers [12], tuberculosis [13], transplantation [14], eclampsia [15] and autoimmunity [16]. IL-10 is a key regulatory cytokine produced in innate and adaptive immune responses that is capable of inhibiting both Th1 and Th2-like cytokine responses, a variety of immune effector mechanisms and expression of inflammatory or autoimmune disorders [17]. Enhanced or differential IL-10 production has been linked to clinical tolerance in animal models and humans in multiple systems [18], [19]. These observations have led several groups to seek evidence of differential systemic serum or plasma IL-10 levels as biomarkers in conditions ranging from pre-eclampsia and systemic lupus erythematosus [15], [16] to food allergy [20].

In a recent clinical study involving 12 DBPCFC-negative subjects and 29 adults who were clinically allergic to hazelnut, Alonso *et al*. [20] proposes the use of constitutive serum IL-10 levels as a marker of clinical tolerance in hazelnut allergy. They report that serum IL-10 levels greater than 2.28 pg/ml were diagnostic of clinical tolerance, with this approach exhibiting sensitivity of 82% and specificity of 70%.

While IL-10 is clearly an important component of immune regulation, given the wide variety of innate and adaptive immune cells that produce IL-10, the extensive range of environmental stimuli and the many individual antigens to which humans are exposed daily, it seemed unlikely to us that constitutively elevated IL-10 levels would provide a useful tool to discriminate between clinical tolerance and food allergy to any single antigen. Here, in more than 200 adults and children who were either clinically tolerant or clinically allergic to peanut, we quantified endogenous IL-10 levels to assess the potential utility of elevated IL-10 as a reliable biomarker of clinical tolerance to peanut.

## Methods

### Ethics statement and Study populations

This study was approved by the University of Manitoba Research Ethics Board. Before enrolment, written informed consent was obtained from 56 adults and from parents of 145 children. Subjects were divided into study groups based on their age and clinical reactivity to peanut. Peanut allergic subjects (History positive/SPT positive: Hx+SPT+) had anaphylaxis upon peanut exposure within the past 5 years, had peanut skin prick tests (SPT, following epicutaneous testing with 1∶10 wt/vol peanut/glycerin extract (Hollister-Steir, Spokane WA) of >3 mm wheal in comparison to the negative saline control and frequently had peanut specific ImmunoCAP values >0.35 kUa/L. Many of these peanut allergic subjects also had positive skin tests or clinical symptoms to additional unrelated allergens. 94 subjects were recruited fitting this definition. One hundred and seven clinically tolerant, unsensitized subjects (History/SPT negative: Hx−SPT−) were recruited on the basis of a detailed history consistent with a lack of clinical reactivity to peanut, negative skin prick tests to peanut, and peanut specific IgE ImmunoCAP results <0.35 kUa/L. These participants took no precautions to avoid peanut, and ingested it without developing any symptoms or signs.

### Plasma IL-10 quantification

Fifty and 20 ml of peripheral blood were obtained by venipuncture from adults and children, respectively. Plasma was isolated by centrifugation. Human plasma IL-10 cytokine levels were quantified in an assay optimized to provide sensitivity <5.0 pg/mL with interassay variability of 5–10% (BioLegend San Diego, CA) essentially as described in Stefura *et al*. [21] but using JES3-9D7 as capture reagent. Statistical analysis was performed using Prism 5 (GraphPAD Software, San Diego, CA).

## Results

**Figure 1:**
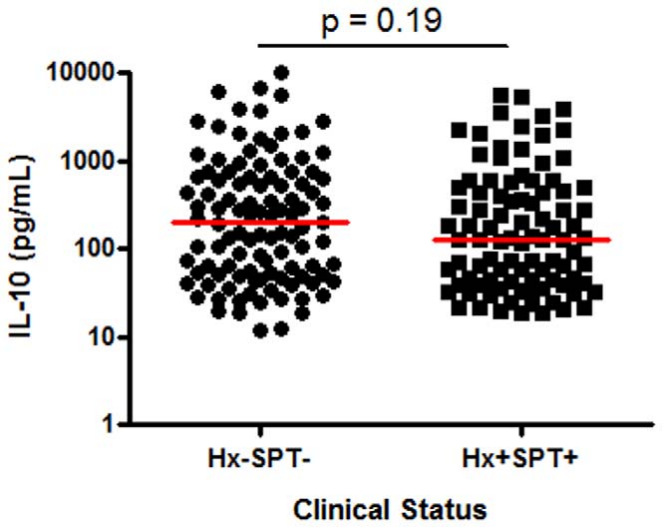
Clinically peanut-tolerant individuals do not exhibit higher plasma IL-10 levels. Clinically peanut-tolerant (history −, SPT −, circles, n = 107) and peanut-allergic (history+, SPT+, squares, n = 94) individuals are shown with bars indicating median values.

The median ages and range of the study populations were as follows: control children (13, 12–15 y), control adults (29, 18–46 y), peanut allergic children (10, 1–17 y) and adults (31, 18–56 y). To determine if plasma IL-10 differed significantly between populations who were clinically tolerant to peanut versus those exhibiting clear clinical histories of symptoms and signs after peanut ingestion, positive SPTs to peanut allergen, and elevated peanut specific IgE, the plasma from each group was analyzed to quantify constitutive IL-10 levels using a high sensitivity ELISA. No difference was evident in the frequency of constitutive IL-10 producers between clinically tolerant and peanut allergic populations. IL-10 was detectable in all 201 subjects, with levels ranging 12 to 9860 pg/ml. Moreover, no quantitative difference in IL-10 levels was evident between the two clinical groups (median response: Hx−SPT−, 197 pg/ml; Hx+SPT+, 128 pg/ml, p = 0.2) (**Figure 1**).

**Figure 2:**
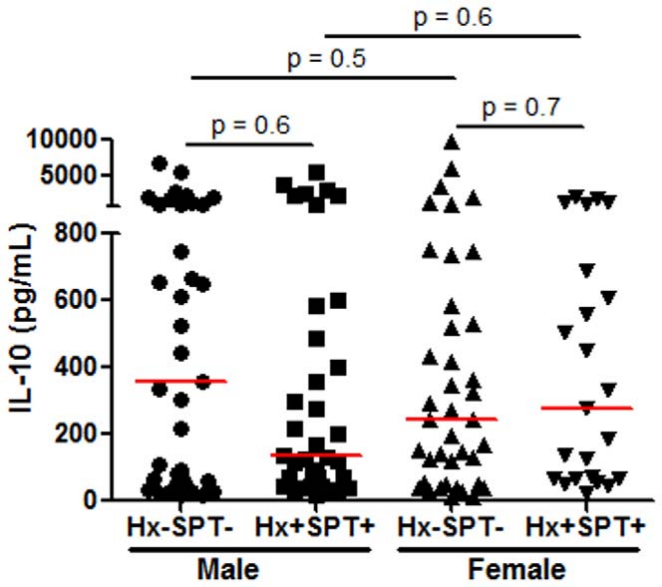
Gender differences do not reveal significantly different plasma IL-10 levels between children who were clinically tolerant to peanut and those who were peanut-allergic. Male (history − SPT−, n = 37; history+, SPT+, n = 37) and female (history − SPT−, n = 40; history+ SPT+, n = 23). Bars indicate medians.

Food allergy prevalence is strongly influenced by age and gender. Therefore, we carried out a subanalysis stratifying the populations to test the hypothesis that in vivo IL-10 levels could provide a useful cytokine biomarker to diagnose clinical tolerance to peanut within specific at-risk groups. The putative effect of gender on IL-10 levels in the two phenotypes was analyzed, as shown in **Figure 2**. Plasma IL-10 levels did not differ significantly between peanut-tolerant and peanut allergic adults in men or in women, data not shown). Similarly, plasma IL-10 levels did not differ significantly between peanut tolerant and peanut allergic children, either in males (p = 0.6) or in females (p = 0.7). Thus within both pediatric and adult, male or female, populations no differences can be detected between plasma IL-10 levels of peanut allergic vs peanut-tolerant individuals.

**Figure 3:**
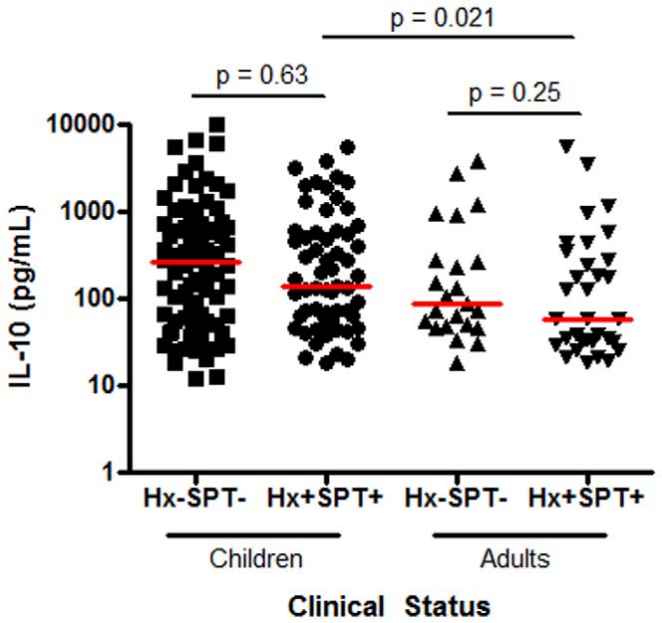
Persistent peanut allergy is associated with lower plasma IL-10 levels. Clinically peanut-tolerant (history − SPT−) (children, n = 84; adults, n = 23) and peanut-allergic (history+, SPT+) (children, n = 61; adults, n = 33) are compared.

Given that peanut allergy, unlike most food allergies, is typically life long, we also sought evidence of age-related differences in plasma IL-10 levels (**Figure 3**). As seen above, plasma IL-10 levels were not significantly different between adults who were clinically tolerant to peanut and those who were clinically allergic (p = 0.25). Plasma IL-10 levels were also indistinguishable between children who were clinically tolerant to peanut and those who were clinically allergic to peanut (p = 0.63). These data indicate that this putative biomarker was not inherently more useful in one specific age group versus another. Plasma IL-10 levels were not linked to age in this cross sectional analysis when comparing clinically tolerant adults and children (p = 0.23). However, interestingly, plasma IL-10 levels were significantly lower in peanut-allergic adults relative to peanut-allergic children (p = 0.021).

## Discussion

We report that elevated plasma IL-10 levels, suggested as a useful biomarker of clinical tolerance to tree nuts in a study of 41 subjects [20], do not successfully discriminate between those with convincing histories of clinical reactions to peanut vs those who ingest and tolerate peanut as part of their usual diet. In this population of 201 subjects, we also conducted sub-analyses targeting the potential utility of this biomarker in specific at-risk groups based on age or gender. None of these analyses supported the hypothesis that elevated plasma IL-10 levels provide a useful diagnostic tool to help identify clinical tolerance to peanut.

In many countries, peanut allergy poses the greatest risk of anaphylaxis of all food allergies [1], [22]. At the same time, the frequency of sensitized but clinically tolerant individuals in the population greatly exceeds that with clinical peanut allergy, emphasizing the need for better tests to complement skin prick tests and measurement of specific IgE levels in positively identifying food allergy. Oral peanut challenges are costly, time-consuming and are not without risk [6]. They are also performed under optimal clinical conditions; for example, a challenge would not be performed if a patient had an acute infection, even an upper respiratory tract infection, or if a patient had asthma symptoms on the day of the challenge. As such, they are not completely predictive of clinical reactivity in the real world. These and other factors limit their use, particularly in an allergy practice setting [22], [23]. Both skin testing and ImmunoCAP peanut specific IgE data yield large proportions of individuals in the “gray” areas characterized by poor positive and negative predictive values.

Reports suggesting that serum IL-10 might act as a biomarker for clinical tolerance are reasonable based on extensive studies in murine and human systems. IL-10 is capable of suppressing production of Th1 and Th2 associated cytokines, and there is extensive evidence suggesting a role for elevated IL-10 in immune tolerance [18], [19], [22], [24], [25]. It is important to note however, that most of the studies that suggest a role for elevated IL-10 levels in tolerance are derived from antigen-specific systems rather than from analysis of systemic IL-10 levels which we believe are more likely to reflect the net immune response of the organism to the millions of immunogenic stimuli encountered. In our well-powered population, constitutive plasma IL-10 levels did not provide a useful marker of clinical tolerance to peanut. Similarly, sub-analyses stratifying by age also failed to demonstrate utility for elevated plasma IL-10 levels in assessing clinical tolerance. Interestingly, among peanut allergic, but not peanut-tolerant subjects, lower plasma IL-10 levels were found to be associated with age, median levels falling by half from 140 pg/ml to 60 pg/ml (p = 0.021). Summers et al in a retrospective, cross-sectional analysis of over 1000 patients to determine which clinical and laboratory parameters best predict the likelihood of severe allergic reactions found enhanced significant relationships between age (adults vs. children) and increased upper, lower respiratory tract or systemic symptoms of anaphylaxis [26]. An important caveat to interpretation of such results is that these studies are cross-sectional rather than longitudinal. Nevertheless, collectively the findings raise the speculative possibility that with long term persistence of food allergy, the capacity of peanut allergic patients to reverse or “grow out of” their clinical reactivity to peanut may be associated with inherently inferior anti-inflammatory IL-10 production. Prospective longitudinal studies will be required to definitively address this question.

Subanalyses focussed on gender also failed to demonstrate utility for this approach in diagnosis of clinical tolerance to peanut. While recent evidence shows that the severity of symptoms elicited by peanut or tree nut anaphylaxis does not differ by gender [26], there is longstanding epidemiological evidence that while males clearly dominate in early life, the prevalence of food allergies and anaphylaxis overall is higher in females, with a 60∶40 ratio for female to male patients [[Review, 27]]. Alternatively others argue that the diagnosis of anaphylaxis can be frequently missed in young infants or children who cannot describe their symptoms. Fatality series focused on food-triggered anaphylaxis do not show a strong female predominance [1]. Here, stratification by gender fails to reveal differences or trends in plasma IL-10 levels between, or within, clinical phenotypes.

In the past, attempts at using biomarkers that are not specific to the disease or specific allergen(s) in question have often shown a lack of usefulness in predicting future events. Total IgE was once measured for the diagnosis of allergy, until it was shown that these levels are poorly predictive of clinical status because they vary substantially depending on gender, race, and other variables, making decision point cut-offs impossible to establish [28]. Elevated serum biomarkers may have a tenuous correlation with allergen-driven responses, making their use difficult [29]. Interestingly, recent studies which aimed to use serum IL-10 as a biomarker in drug allergy found no difference between IL-10 plasma levels in clinically tolerant vs. penicillin-allergic subjects [30]. Sharply elevated, rather than reduced, IL-10 levels were evident amongst 36 patients who presented to emergency departments with severe acute anaphylaxis episodes [31]. These results emphasize how challenging identification of validated antigen non-specific diagnostic biomarkers will be.

Among allergen-specific biomarkers, peanut specific IgE levels are useful for identification of patients at lowest risk of clinical reactivity to peanut (levels <0.35 kUa/L, absent or undetectable) and for identification of those at highest risk for clinical reactivity to peanut (levels ≥14 kUa/L (100% PPV, 95% C.V.). However, they are less useful in the many peanut-sensitized patients who have values in the range of 0.35–14 kUa/L [4], [5]. Skin prick test diameter can be useful in predicting the likelihood of clinical reactivity to peanut, but as with peanut specific IgE levels, this approach is greatly hampered by poor specificity and poor positive predictive values in a substantial proportion of the population [7]. In addition, neither peanut SPT nor peanut specific IgE levels reliably predict the severity of the clinical response. Peanut SPT and specific IgE levels may remain positive years after clinical tolerance towards peanut has been achieved [22].

One limitation in the study design used here is that DBPCFC were not performed on the 200 subjects for ethical and practical reasons. Peanut non-allergic subjects reported that they regularly ate peanut. Diagnoses of peanut allergy were made by experienced pediatric allergists based on recent convincing histories of peanut induced anaphylaxis, peanut specific IgE, and peanut skin prick test results. While some misclassification is possible, in our view, the overlap in range of IL-10 values between the populations, spanning almost three orders of magnitude, makes it extremely unlikely that this parameter would serve as a useful diagnostic marker of clinical tolerance with acceptable positive and negative predictive values.

The primary focus in this study was testing the hypothesis that globally enhanced plasma or serum IL-10 levels could provide a sensitive and specific tool for determination of human clinical tolerance to food antigens. For peanut allergy it did not. The mechanisms that are responsible for ongoing clinical tolerance to peanut in the 97–99% of the general population that is not clinically allergic to peanut remain unclear. Thus, the broader question of what combination of mechanisms is responsible for establishing and maintaining oral tolerance to foods in most of the human population remains controversial. Evidence suggests the participation of multiple mechanisms, including elevated antigen-specific (rather than global) IL-10, TGFb and/or IL-35 production, antigen-driven CTLA4 expression, alterations in tryptophan metabolism, anergy of food-specific T cells and activity of one or more of the 15 regulatory T cell subsets putatively identified to date [32], [33].

In summary, we demonstrate that constitutive plasma IL-10 levels do not serve as a useful biomarker to discriminate clinical tolerance to peanuts from peanut allergy in humans. Further research is required to identify clinically useful markers by distinguishing factors that differ quantitatively between those who are peanut allergic and those who ingest peanut without allergic reactions. With greater understanding of how tolerance is achieved and maintained, identification of such biomarkers may be possible.
